# Microbiome Signatures in Advanced Gastric Cancer: Emerging Biomarkers for Risk Stratification, Therapy Guidance, and Prognostic Insight

**DOI:** 10.3390/ijms27031452

**Published:** 2026-01-31

**Authors:** Kyung-il John Kim, Hannah Zhong, Derek Tai, Pranati Shah, Daniel Park, Vitor Goes, Jianan Li, Claire Jung, Lucas Kim, Sofia Guzman, Gagandeep Brar, Dani Castillo

**Affiliations:** 1 Department of Internal Medicine, Kaiser Permanente Fontana Medical Center, 9961 Sierra Ave., Fontana, CA 92335, USA; kyungil.j.kim@kp.org; 2 Department of Hematology and Hematopoietic Cell Transplantation, City of Hope, 1500 E Duarte Rd., Duarte, CA 91010, USA; hazhong@coh.org; 3 Department of Internal Medicine, Loma Linda University Medical Center, 11234 Anderson St., Loma Linda, CA 92354, USA; dtai@llu.edu (D.T.); pdshah@llu.edu (P.S.); 4 Department of Hematology and Oncology, Harbor-UCLA Medical Center, 1000 W Carson St., Torrance, CA 90502, USA; dparkl7@dhs.lacounty.gov; 5 Department of Internal Medicine, Hospital Israelita Albert Einstein, Av. Albert Einstein, 627-Morumbi, São Paulo 05652-900, SP, Brazil; vitor.goes@einstein.br; 6 Faculty of Kinesiology & Physical Education, University of Toronto, 100 Devonshire Place, Toronto, ON M5S 2C9, Canada; jianan.li@mail.utoronto.ca; 7 Flintridge Preparatory School, 4543 Crown Ave., La Cañada Flintridge, CA 91011, USA; 28jungc@flintrridgeprep.org; 8 School of Medicine, Loma Linda University, 11175 Campus St., Loma Linda, CA 92350, USA; lucaskim@students.llu.edu; 9 Department of Medical Oncology and Therapeutics Research, City of Hope, 1500 E Duarte Rd., Duarte, CA 91010, USA; sofguzman@coh.org

**Keywords:** gastric cancer, advanced gastric cancer, gastric microbiome, biomarkers

## Abstract

Gastric cancer (GC), often diagnosed at advanced or metastatic stages, remains a significant clinical challenge requiring novel biomarkers for early detection, risk stratification, and effective, personalized treatment optimization. Emerging evidence underscores a strong association between gut microbiome dysbiosis and GC initiation, progression, and therapeutic outcomes. This review explores the potential of the advanced/metastatic gastric microbiome as a source of diagnostic and targetable biomarkers and its role in modulating responses to immunotherapy. Although *Helicobacter pylori* (*H. pylori*) is the most significant risk factor for GC, several other gastrointestinal taxa—including *Fusobacterium nucleatum* (*F. nucleatum*)—have been implicated in advanced GC (AGC). At its inception, microbial dysbiosis contributes to chronic inflammation and immune evasion, thereby influencing tumor behavior and treatment efficacy. Integrating microbiome-based biomarkers into risk stratification, GC staging, and targetable treatment frameworks may enhance early detection, inform immunotherapy strategies, and improve patient-specific treatment responses. *Bifidobacterium* and *Lactobacillus rhamnosus GG* have the potential to change the immunotherapy framework with their direct influence on dendritic cell (DC) and cytotoxic T cell (CTL) activity. However, clinical translation is impeded by methodological heterogeneity, causality limitations, and a lack of clinical trials. Nonetheless, the integration of microbiome profiling and the development of therapeutic microbiome modulation strategies, such as personalized probiotics regimens and fecal microbiota transplantation, hold substantial potential for improving clinical outcomes and reducing treatment-related toxicity in GC management.

## 1. Introduction

Gastric cancer (GC) currently ranks third in the global causes of deaths related to cancer, with close to 783,000 deaths each year [1,2]. As the fifth-most-diagnosed malignancy, nearly one million new incidents are reported every year, with nearly half the cases presenting in advanced or nonresectable stages due to a lack of early signs and symptoms [2,3]. The incidence of advanced gastric cancer (AGC) is highest in the Eastern/Central Asia and Latin America regions, primarily driven by shared risk factors including a higher prevalence of *Helicobacter pylori (H. pylori)* infection, dietary lifestyles, alcohol consumption, and socioeconomic disparities.

The gastrointestinal (GI) microbiome has been vastly overlooked in the pathophysiology of AGC. Pathogenic alterations of the gastric microbiota, known as dysbiosis, reduce the diversity of commensal microorganisms and result in decreased tumor immunity, with pathogenic alterations to the gut microbiome. Identifying and characterizing the functional profiles of altered bacterial taxonomic groups in GC patients holds promise in deriving novel diagnostic and targetable biomarkers, suggesting its emerging role as a valuable predictive tool in precision oncology. *Bifidobacterium* and *Lactobacillus rhamnosus GG*, for example, are emerging biomarkers with remarkable tumor-suppressive properties, with recent studies highlighting their role in enhancing immune checkpoint inhibitor therapy. These microbes are frequently observed to be downregulated in GC and are one of a few biomarkers with incredible prospective value in clinical metastatic GC treatment. However, even with the emergence of these novel biomarkers, further investigation is needed before their implementation into actual practice. The objective of this review is to examine bacterial biomarkers within the GI microbiome associated with GC that may serve as predictive markers for the development of metastatic/nonresectable GC, and treatment responses to chemotherapy and immunotherapy, and for recognizing pathologic microbial changes throughout the clinical course, where GI microbiome optimization via probiotic supplementation or fecal transplantation may improve patient outcomes. By integrating microbiome profiling into existing clinical frameworks, we aim to propose a more comprehensive staging approach and evaluate the potential modulatory effects of the microbiome on disease identification, progression, and treatment efficacy in AGC.

## 2. Key Microbial Signatures That Influence the GC Tumor Microenvironment (TME)

Patients with GC are characterized by gut microbial dysbiosis with significant alterations of up to 52 bacterial genera compared to patients with precancerous lesions [4,5,6]. As noted in Table 1, the most notable alterations include intratumoral or fecal enrichment in *Fusobacterium*, *Streptococcus*, *Lactobacillus*, and *Prevotella* and depletion in *Bifidobacterium* [5,6,7,8]. Identifying the pattern of alterations of these microbial signatures as gastric tissue evolves through Correa’s cascade offers valuable insight into their diagnostic and targetable roles in GC management.

### 2.1. Fusobacterium Activates the Pro-Inflammatory IL-17/NF-κB/RelB and PI3K/AKT Signaling Cascade

An emerging non-*H. pylori* bacterium gaining notoriety as a potential targetable biomarker is *Fusobacterium nucleatum* (*F. nucleatum*), a Gram-negative anaerobic gut commensal [12,13,14]. Current literature supports that *F. nucleatum*’s abundance in GC is directly related to *H. pylori* status. *F. nucleatum* is enriched throughout in *H. pylori-*negative (*HP-*negative) GC cases; however, this pathogen’s presence is masked in the early stages of *H. pylori-*positive (*HP-*positive) cases when *H. pylori* dominates the gut as it initiates Correa’s cascade [14]. As *H. pylori*’s relative abundance begins to dwindle with the gastric epithelium assuming a metaplastic or dysplastic phenotype, the opportunistic *F. nucleatum* proliferates within the gut as it maintains the pro-inflammatory microenvironment established by *H. pylori*. *H. pylori* and its virulence factors contributing to GC are extensively studied, with its eradication being the only population-based measure, per American Gastroenterological Association (AGA) guidelines, to prevent GC in the United States [15]. However, pre-clinical studies demonstrating *F. nucleatum*’s potent role in maintaining a pro-inflammatory TME offer novel targets for GC prevention and treatment.

**Figure 1:**
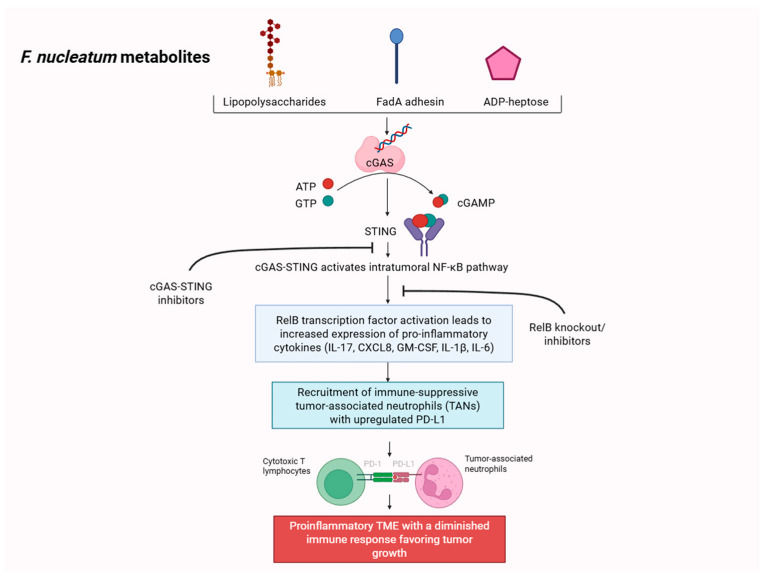
*F. nucleatum* induces NF-κB signaling with the production of pro-inflammatory cytokines, leading to recruitment of tumor-associated neutrophils (TANs) expressing PD-L1, attenuating cytotoxic T lymphocytes’ activity. Created in Biorender. Kyung-il John Kim. (2026) https://app.biorender.com/illustrations/696eea17cd44920b10c0314f?slideId=ced5d395-8a86-4f56-bb8e-f08a67a41157, accessed on 21 January 2026.

*F. nucleatum* directly contributes to GC progression by creating a pro-inflammatory and immune-evasive TME by activating the intratumoral IL-17/NF-κB/RelB signaling pathway as depicted in Figure 1 [7,16]. Although *F. nucleatum*-specific antigens that activate this pathway in GC are not clearly established, pre-clinical studies that analyzed this pathway in CRC suggest that *F. nucleatum*-derived lipopolysaccharides, FadA adhesin, and ADP-heptose are primarily responsible for activating this cascade [17,18,19,20]. Downstream effects of this cascade result in upregulated expression of pro-inflammatory cyto-/chemokines including TNF-α, CXCL8, CM-CSF, IL-1β, and Il-6 [7,21,22,23,24]. Though less studied in GC, these inflammatory markers lead to recruitment of tumor-associated neutrophils (TANs), which acquire the immune-suppressive phenotype with increased expression of PD-L1, dampening the cancer-killing effects of cytotoxic T lymphocytes (CTLs) [7].

Zhang et al. demonstrated that *F. nucleatum*-induced upregulation of PD-L1 expression from TANs was reversable with direct inhibition of the cGAS-STING pathway and RelB knockout [7]. Integral to the host immune response, the cGAS-STING mechanism plays crucial roles in both innate and adaptive immunity. Therefore, inhibition of the cGAS-STING pathway, upstream of IL-17/NF-κB, resulting in restored immunity suggests its context-dependent role in the setting of a *F. nucleatum*-induced dysregulated immune TME. In addition, knockout of RelB, a transcription factor for pro-inflammatory cyto-/chemokines, in GC cells led to significantly reduced IL-17 and recruitment of TANs [7]. With consideration that first-line treatment for advanced GC includes PD-1 inhibition plus CT, *F. nucleatum*’s ability to upregulate PD-L1 poses significant implications as a novel targetable pathway in GC.

Furthermore, *F. nucleatum*’s role in GC progression is further elucidated by its role in activating the oncogenic PI3K/AKT signaling pathway by modulating the contents of exosomes secreted by *F. nucleatum*-infected GC cells. Xin et al. demonstrated that, in vivo, *F. nucleatum*’s infiltration of GC cells was shown to increase exosomal HOTTIP (HOXA transcript at the distal tip) levels [22]. Translocation of HOTTIP-enriched exosomes into F-nucleatum-absent GC cells subsequently promoted tumor growth and metastasis [25]. HOTTIP, a long noncoding RNA, acts as a competing RNA and binds to miR-885-3p, suppressing its inhibition of EphB2 expression. Increased levels of EphB2, a tyrosine kinase receptor, leads to increased activation of the oncogenic PI3K/AKT signaling cascade [26,27].

*F. nucleatum*-induced activation of the IL-17/NF-κB/RelB and PI3K/AKT signaling pathways contributes to a diminished immune response and pro-inflammatory TME, a potential target for GC treatment that remains vastly unexplored. Future studies targeting these signaling pathways should consider assessing the GC tumor burden and metastasis in animal models.

### 2.2. Prevotella Upregulates GC-Perilipin 3 (PLIN3)

*Prevotella*, an anaerobic Gram-negative rod, is linked with chronic inflammatory disease (e.g., periodontitis, inflammatory bowel disease [IBD], rheumatoid arthritis); its pro-inflammatory properties and enrichment in GC tissue suggest its direct role in GC development [28,29]. A pre-clinical study by Liang et al. discovered that *Prevotella intermedia* (*P. intermedia*) directly contributes to GC progression by upregulating perilipin 3 (PLIN3) in GC cells [27]. In vitro, GC cell lines were treated with intratumoral-derived *P. intermedia* supernatant, with the results showing a much more aggressive and malignant phenotype with increased cellular proliferation, migration, and invasion [9]. In order to identify dysregulated proteins in GC cells post-exposure to *P. intermedia* supernatant, label-free protein analysis identified significant upregulation of PLIN3. As a scaffold protein, PLIN3 essentially contributes as a metabolic stress buffer and ensures the survival and adaptability of cancer cells by regulating the formation of and stabilizing intracellular lipid droplets. The tumor-promoting properties of this protein were supported by its shRNA-mediated knockout, which abrogated its effects [9].

As there are no other pre-clinical/clinical studies to date that target PLIN3, future studies should focus on the development of novel agents that target GC cell PLIN3 in animal models with potential for clinical application in treating GC in human patients.

### 2.3. Fusobacterium and Prevotella Can Predict GC Aggressiveness

The previous sections discussed the mechanisms in which both *Fusobacterium* and *Prevotella* directly contribute to GC development with potential targets for treatment; however, these two genera may also serve as prognostic biomarkers that predict tumor aggressiveness [30]. A cohort study by Lehr et al. demonstrated that intratumoral enrichment of *Fusobacterium* and *Prevotella* was correlated with more aggressive tumor phenotypes and poorer clinical outcomes [30]. Paired tumorous (T-GC) and adjacent non-tumorous tissues (NT-GC) from 64 patients (*n* = 128) with GC were biopsied and analyzed via 16S rRNA sequencing, which yielded 19 unique phyla and 296 genera which were correlated with patient survival via Cox regression models. Analysis showed elevated relative abundances of *Fusobacterium* and *Prevotella* associated with poorer overall survival [28].

Subgroup analysis was performed based on Lauren’s classification, an established histopathological framework of GC which yet remains underutilized in microbiome-focused research. While no significant survival associations were noted in the intestinal-type subgroup, diffuse-type GC patients with *F. nucleatum*-positive tumors exhibited significantly worse overall survival [30]. To better understand the contribution of *F. nucleatum* to GC development, the study extended its analysis to include normal gastric mucosa, as well as samples with chronic non-atrophic gastritis (CNAG), atrophic gastritis, and intestinal metaplasia (AG/IM). The presence of *F. nucleatum* was then correlated with clinicopathologic features and long-term outcomes, further supporting its potential role as a targetable biomarker in the diffuse-type GC subtype [30]. Although the intestinal subtype per Lauren’s criteria is primarily driven by Correa’s cascade, the diffuse subtype is more genetically driven, explained by a germline mutation in the E-cadherin (CDH1) gene resulting in poor cohesion between epithelial cells in the gastric mucosa, increasing the risk of tumor invasion and peritoneal involvement [31]. Therefore, incorporating *Prevotella* and *Fusobacterium* into existing predictive and prognostic models may enhance the precision and confidence of risk stratification and guidance in the next steps in management.

### 2.4. Streptococcus Induces the Pro-Inflammatory ANXA/MAPK and Gasdermin E (GSDME)-Mediated Pyroptosis Pathway

As a microbe responsible for common infections such as strep-throat, pneumonia, and soft-tissue infections, certain strains of *Streptococcus* (*Streptococcus anginosus* [*S. anginosus*] and *Streptococcus constellatus* [*S. constellatus*]) may directly contribute to GC development by influencing the TME and accelerating Correa’s cascade [32]. In addition, multiple studies consistently report this genus to be enriched in *HP*-negative/-positive GC tissue and fecal samples and, when enriched with *Lactobacillus*, to have GC stage diagnostic power with area under the curve (AUC) = 0.7949; this was validated in an independent cohort with AUC = 0.7712 [32,33,34].

Mechanistically, *S. anginosus* infection in murine models has been shown to induce acute gastritis and accelerate carcinogenesis by upregulating activation of the MAPK signaling cascade. *S. anginosus* colonization of gastric epithelium is mediated by the expression of *Trepenoma pallidum* membrane protein C (TMPC), which binds to the gastric epithelial Annexin A2 (ANXA2) receptor, activating the MAPK signaling pathway [35]. Downstream effects of the MAPK cascade ultimately result in upregulated cellular proliferation and production of pro-inflammatory cytokines, effectively accelerating Correa’s cascade towards the dysplastic/carcinoma stages [35,36].

*S. anginosus* further induces and maintains a pro-inflammatory TME by activating the GSDME-mediated pyroptosis pathway. Pyroptosis, otherwise known as inflammatory cell death, plays a crucial role in regulating cell survival. In the setting of an acute infection, acute pyroptosis stimulates immune cell recruitment to areas of infection and eliminates damaged cells. However, in vivo and in vitro studies demonstrated that persistent and dysregulated activation of GSDME by cleaved caspase-3 and NLRP3 (NOD-like receptor family pyrin domain containing 3) leads to excess and prolonged release of pro-inflammatory cytokines, conferring a tumor-conducive microenvironment [5,37].

Pre-clinical studies utilizing MAPK inhibitors in GC murine models have been shown to mitigate the pathway’s tumor-promoting effects, with reduced inflammatory markers, cellular proliferation, and tumor migration/invasion [37]. Meanwhile, no studies to date have targeted *S. anginosus*-mediated GSDME activation and its downstream pyroptosis pathway. Within the broader scope of GC risk stratification and treatment, *Sreptococcus* shows promise as a diagnostic and targetable biomarker. Future studies should investigate the effects of MAPK inhibitors in human GC patients enriched with *Streptococcus* and develop inhibitors targeting the GSDME-mediated pyroptosis pathway.

### 2.5. Lactobacillus and Its Dual Role in GC

*Lactobacillus*, a Gram-positive lactate-producing bacilli, is a popular ingredient in modern-day probiotics to support gut health; however, recent studies reporting enrichment of *Lactobacillus* in GC patients emphasize strain-specific variation in function [5,9,10]. As a lactate-producing genus, the pro-tumorigenic properties of *Lactobacillus* stem from their preference for utilizing glycolysis as the primary source of energy production in tumor cells, also known as the Warburg effect [38]. First described by Zhang et al., post-translational histone lactylation of lysine residues in vitro driven by increased lactate upregulates chromatin gene transcription [39]. Implicated in GC, upregulation of arginase 1 (Arg1) expression in macrophages promotes the transition of M1 tumor-associated macrophages (TAMs) to the tumor-conducive M2 TAM phenotype [39,40]. The pro-tumorigenic M2 TAMs contribute to an immune-evasive TME by increasing the secretion of immunosuppressive cytokines (IL-4, IL-10, IL-13, IL-1RA, TGF-β) and facilitate the epithelial-to-mesenchymal transition (EMT) with growth factors and matrix metalloproteinases [41,42,43].

Pre-clinical studies targeting lactate production with lactate dehydrogenase A (LDHA) inhibitors and histone lactylation with inhibition of the respective transferase (p300/CBP) in murine models have shown inhibition of their tumor-promoting effects in pancreatic cancer and glioblastoma, respectively [44,45]. However, these targets and effects in GC animal models with enriched *Lactobacillus* have not yet been studied.

Although current literature suggests *Lactobacillus* as a contributor to GC development, specific strains of this genus (e.g., *Lactobacillus rhamnosus GG* [*L. rhamnosus GG*], *Lactobacillus gasseri* [*L. gasseri*]) display remarkable antagonistic effects against *H. pylori*-induced tumorigenesis in vivo and in vitro. *L. gasseri*, for example, inhibits *H. pylori* cytotoxin-associated gene A (CagA) phosphorylation, preventing its interaction with host Src homology-2 protein tyrosine phosphatase (SHP-2) [46]. This mitigates downstream pro-inflammatory effects such as NF-κB activation and upregulated release of IL-8, an inflammatory cytokine involved in intestinal metaplasia transformation [47,48]. Additionally, both *L. gasseri* and *L. rhamnosus GG* have been shown to reduce EMT by directly decreasing the expression of EMT-inducing transcription factors, Snail and Zeb-1. As Snail and Zeb-1 block transcription of E-cadherin, inhibition of Snail and Zeb-1 by *L. gasseri* and *L. rhamnosus GG* reduces the loss of epithelial integrity and subsequent migration and invasion of tumor cells [49,50]. Specific strains of *Lactobacillus* such as *L. rhamnosus GG* and *L. gasseri* show promise as personalized therapy to optimize the GI microbiome in GC. However, as these beneficial effects are primarily observed in animal models, future studies should focus on human applications to assess the tumor burden with *L. rhamnosus GG* and *L. gasseri* supplementation.

### 2.6. Bifidobacterium Directly Inhibits H. pylori-Induced miR-145 Expression and Enhances Dendritic Cell (DC)-Mediated Immunity

*Bifidobacterium* is widely considered to be a protective genus in the human gut, with its depletion associated with an increased risk of *H. pylori-*induced ulcers and tumor transformation. The anti-tumor effects of *Bifidobacterium* are multi-modal, with substantial ability to enhance the immune response to cancer cells, promote apoptosis, and inhibit *H. pylori*-mediated carcinogenic pathways [51].

The anti-tumorigenic properties of *Bifidobacterium* supplementation were initially tested in vitro with *H. pylori-*infected human gastric epithelial cell lines (GES-1) [51]. Through NGS analysis of cell lysates, investigators determined *H. pylori-*induced elevation of microRNA (miR)-145 as a positive regulator of the Wnt/β-catenin and COX-2 signaling pathways, which promote proliferative and metastatic changes [52]. However, *Bifidobacterium lactis (B. lactis)* supplementation significantly reduced the expression of miR-145 in a dose-dependent fashion, with suppressed Wnt/β-catenin and COX-2 signaling. With these results, the investigators administered *B. lactis* probiotics to *HP-*positive patients with IM (*n* = 48) and observed a significantly higher rate of IM regression compared to control [52].

*Bifidobacterium* also demonstrates the unique ability to enhance the host immune response against GC [53]. This genus directly promotes dendritic cell (DC) maturation, with the subsequent release of cytokines conducive to cytotoxic T-cell and natural killer (NK) cell activity against tumor cells [54,55,56,57]. Butyrate is a short-chain fatty acid (SCFA) and a natural metabolite produced by *Bifidobacterium* that has also demonstrated immune regulatory properties by reducing peripheral mononuclear cell expression of PD-L1 and IL-10, optimizing effector and regulatory cell functions [58]. In vivo studies demonstrate that administration of butyrate significantly reduced PD-L1/IL-10 expression and gastric tumor size [58].

Lastly, *Bifidobacterium* has shown to play a key role in regulating colon cancer cell survival in vitro by upregulating expression of the pro-apoptotic Bax and caspases, with downregulation of the anti-apoptotic Bcl-2 [59]. However, its effects on human GC lines have not yet been studied.

*Bifidobacterium* is a hallmark of gut homeostasis and antagonist of *H. pylori-*induced carcinogenesis through the establishment of an anti-inflammatory/proliferative gut microenvironment, enhancement of anti-tumor immunity, and regulation of cell survival [60,61,62]. However, more evidence is needed before its application into the *HP-positive GC* management framework. For example, the histologic-regression effects of *B. lactis* have only been established in IM patients but not in those with GC.

## 3. Inter-Study Variations in Reported Microbial Signatures

There are countless large-scale studies identifying compositional differences in gastric tissue or fecal samples from patients with GC compared to healthy controls; however, the variations in reported microbial signatures altered in GC weaken their potential role as diagnostic/targetable biomarkers. These discrepancies are likely a consequence of study design heterogeneity, including differences in patient demographics, patient selection criteria, statistical analysis, and the application of the validation of cohorts.

There is also a clear lack of distinction between significantly altered and discriminatory signatures in GC. Significantly altered microbes are not specific to the cancerous stage but provide insight into how their populations change through Correa’s cascade. On the other hand, GC discriminatory markers were identified with additional statistical analysis, which provide diagnostic value. For example, as noted in Table 1, Liu et al. [5], Coker et al. [6], and Abate et al. [10] report significant enrichment of *Fusobacterium* in GC tissue, but this was not included in GC discriminatory signatures identified by Liu et al. [5] after application of a backward stepwise algorithm. This implies that *Fusobacterium* abundance likely increases through Correa’s cascade but is not specific to GC staging; Wang et al. [9] reports significant enrichment of this genus in precancerous intraepithelial neoplasia. Additionally, the external validity of these findings is uncertain considering that most existing studies are conducted in East Asia in which GC-contributing risk factors such as diet and lifestyle may significantly differ from those in Latin American populations. Although no large-scale studies consisting of primarily Latin American populations have been published, the EU-LATAM partnered LEGACy observational study is an ongoing endeavor to comprehensively characterize the epidemiology of GC and utilize multi-omics to identify treatment-guiding biomarkers [63]. The choice of sample collection (gastric tissue versus fecal sample) also has clinical implications, in which the more invasive approach with tissue biopsy may be indicated in symptomatic/high-risk patients, while fecal samples may serve as noninvasive screening tools.

As current studies are largely conducted in European and East Asian regions, future studies with aims to identify microbial biomarkers in GC via standard 16S rRNA sequencing should include a representative Latin American patient population, which may be extracted from the LEGACy study to account for differences in *H. pylori* strains, diet, environmental exposures, and socioeconomic disparities. Subgroup analysis should also be considered, with categorization by age, sex, gender, region, sample type (gastric tissue vs. fecal), history of gastric resection or neoadjuvant chemotherapy, and *H. pylori* status, to help individualize risk stratification and guide patient-specific treatment. Countless studies exist with aims to characterize the GC microbiome; however, the lack of regional representation and standardization of study methods serves as a barrier to incorporating our knowledge of the microbiome into GC clinical practice.

## 4. Key Microbial Signatures That Modulate and Predict Responses to Immune Checkpoint Inhibitors (ICIs)

ICIs plus systemic chemotherapy is the first-line treatment for HER2-negative advanced GC/GEJ in PD-L1-positive patients. Although ICIs significantly improve patient OS and PFS, the overall prognosis remains poor, with certain subgroups receiving attenuated benefit. GC and its common sites of metastases including the peritoneum and liver are known to confer an immune-suppressed TME, which may explain patient-specific resistance to immunotherapy [64,65,66,67]. The GI microbiome remains clinically unexplored as an adjunctive treatment in non-surgical HER2-negative AGC patients; however, several pre-clinical/clinical studies have demonstrated the ability of certain microbial signatures to enhance the effects of ICIs.

### 4.1. Bifidobacterium and L. rhamnosus GG Enhance ICI by Inducing Type I Interferon (IFN) Signaling

**Figure 2:**
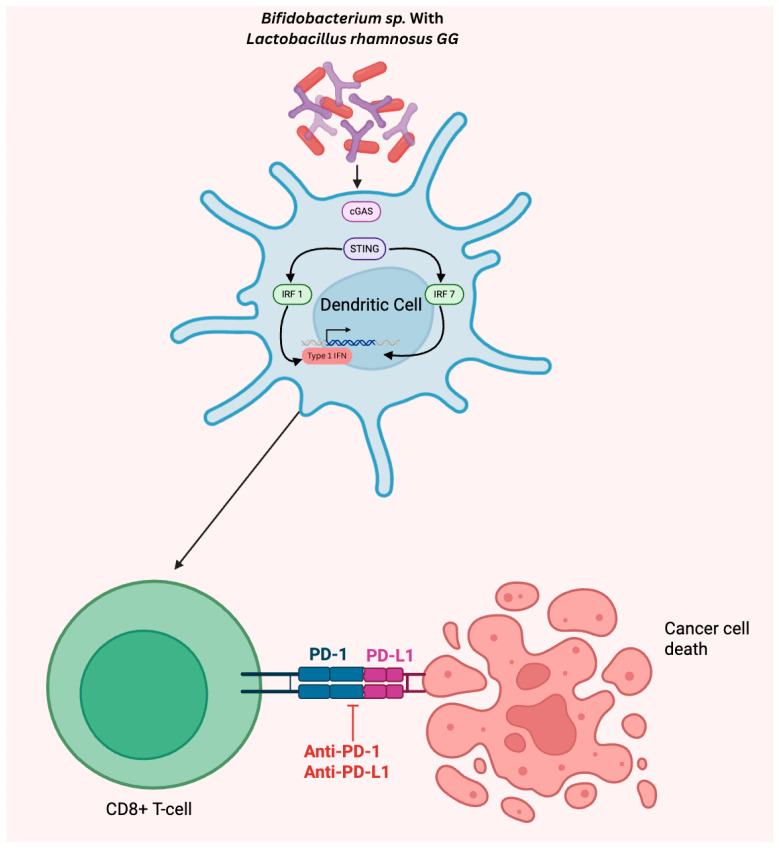
Upregulation of type I IFN signaling by *Bifidobacterium* and *Lactobacillus rhamnosus GG* via the cGAS/STING pathway leading to enhanced CD8+ T cell activity.

*Bifidobacterium* and *L. rhamnosus GG* show immense potential as adjunctive treatment to ICIs in the clinical setting when treating patients with HER2-negative AGC. Mechanistically, in vitro and in vivo studies demonstrate that *Bifidobacterium* and *L. rhamnosus GG* induce a robust immune response by inducing type I interferon (IFN) signaling through the cGAS/STING pathway, with downstream effects leading to the enhanced priming, differentiation, and proliferation of CTLs as depicted in Figure 2 [57,68]. The synergistic effects of *Bifidobacterium* or *L. rhamnosus GG* with ICIs have been studied in murine models with solid tumors such as melanoma, CRC, and NSCLC, with the results demonstrating that oral supplementation of either *Bifidobacterium* or *L. rhamnosus GG* upregulated the tumor infiltration of CTLs and significantly reduced the tumor burden [69,70,71]. However, their effects remain unknown in the setting of GC. In order to further elucidate the synergistic properties of *Bifidobacterium* and *L. rhamnosus GG* with ICIs, future studies should investigate whether these benefits are consistent in murine models with human GC lines receiving oral supplementation of both *Bifidobacterium* and *L. rhamnosus GG* compared to monotherapy alone. Although there are no RCTs evaluating the benefits of *Bifidobacterium* and *L. rhamnosus GG* supplementation in GC patients, one retrospective study determined that probiotic supplementation did not improve outcomes in patients with GC undergoing CT [72]. However, its influence on survival has not yet been studied in patients undergoing ICI treatment.

### 4.2. Microbial Signatures May Predict Response to ICIs in Patients with Advanced GC

GI microbial signatures have the potential to predict immunotherapy responses and may be considered with the current treatment framework in patients with HER2-negative AGC. The DELIVER trial (2021) conducted in Japan was a prospective observational study aiming to determine the efficacy of nivolumab monotherapy in patients with AGC; however, secondary aims included the identification of microbial biomarkers associated with nivolumab response. Metagenomic shotgun sequencing from patient fecal samples identified enrichment of two biomarkers, *Odoribacter* and *Veillonella*, associated with favorable responses to nivolumab, which were validated in a separate cohort [73]. Outside of The DELIVER trial, evidence supporting these two microbial signatures as ICI-predictive markers is limited. In addition, the observational nature of this study precludes the causative vs. correlative role of *Odoribacter* and *Veillonella* in GC and ICI modulation.

The enrichment in responders to ICIs is consistent with a systematic review that identified short-chain fatty acid (SCFA)-producing microbial signatures in human fecal samples associated with favorable ICI responses in patients with various types of cancers [74]. Pre-clinical studies evaluating GC burden in animal models suggest that SCFAs, such as butyrate and propionate, upregulate CD8+ T-cell activity, suppress inflammation by stimulating regulatory T cells, downregulate macrophage PD-L1 and IL-10, and enhance gut epithelial integrity to mitigate tumor invasion and migration [58,75,76]. Although SCFA-producing bacteria have shown to enhance ICI with significantly reduced tumor burden in GC animal models, their immune-augmenting effects in human patients with GC remain unknown. Future studies should focus on prospective trials evaluating tumor burden with a control arm consisting of first-line ICIs plus CT and an intervention arm consisting of combination ICIs plus CT with probiotic supplementation consisting of beneficial SCFA-producing bacteria.

### 4.3. The Potential Benefit of Probiotics Remains Unexplored in Advanced/Nonresectable GC Patients Undergoing ICI

There is limited data that support probiotic supplementation adjunctive treatment in advanced/nonresectable GC patients. The vast majority of studies that have demonstrated benefit with probiotic supplementation have been in patients receiving CT monotherapy, patients in the perioperative window, and patients with treatment-related adverse effects (AEs) [77,78]. Multiple RCTs and prospective studies support probiotic supplementation in GC patients undergoing partial gastrectomy or neoadjuvant CT with minimally invasive surgery, as it has been shown to reduce perioperative complications (infection/inflammation, gut motility recovery) in addition to facilitating earlier hospital discharges and reduced patient costs [77,78,79,80]. However, the lack of evidence demonstrating improved patient outcomes with probiotic supplementation in non-surgical advanced GC patients deters its integration into the overall treatment framework. A meta-analysis evaluating the effects of oral probiotic supplementation in non-surgical GC patients undergoing CT determined that there was no significant benefit in terms of OS/PFS, although the incidence of CT-related diarrhea was reduced [81].

With the emergence of ICIs as part of first-line treatment in advanced GC patients, we believe that *Bifidobacterium* and *L. rhamnosus GG* have not been able to exert their potent immunomodulatory effects in these treated patient populations. Although multiple phase III RCTs (CHECKMATE-649, RATIONALE-305, ATTRACTION-4) show OS/PFS benefits with the addition of ICIs to CT, the overall prognosis remains poor, with close to 25% of patients receiving ICIs experiencing immune-related adverse events (irAEs). Currently, the AGA only recommends probiotics for specific patient populations (e.g., high-risk C. diff infection, necrotizing enterocolitis prevention in infants) and emphasizes that their effects are highly regimen-based [82,83]. With this in mind, future studies should initiate phase I clinical trials to determine the safety profile of varying doses of oral *Bifidobacterium*/*L. rhamnosus GG* in advanced GC patients. This will serve as a crucial step in microbiome-targeted therapeutics, as there have been rare cases of probiotics regimented with *Bifidobacterium/L. rhamnosus* inducing sepsis in neonates and critically ill adults [84,85,86,87,88]. By phase III, OS/PFS and adverse event rates should be re-assessed in all randomized patients and those with positive PD-L1 expression (CPS or TAPs) who are initiating first-line ICIs plus CT.

The immunomodulatory effects of oral probiotics containing *Bifidobacterium*/*L. rhamnosus GG* in nonresectable advanced GC patients undergoing ICI treatment remain unknown. Obstacles needing to be addressed prior to clinical translation are the lack of studies assessing probiotics’ benefit in human GC patients undergoing ICI treatment, standardized probiotics’ dosing and included strains, the consideration of patient-specific microbiome changes, and concerns regarding probiotic-induced infection/sepsis [81,89].

## 5. Future Considerations

The clinical application of microbiome research in GC offers promising avenues for innovation but is accompanied by substantial challenges. A primary limitation is the lack of standardization across methodologies, including sample-collection techniques (e.g., biopsy vs. stool), sequencing strategies (16S rRNA vs. shotgun metagenomics), selection of control groups (SG versus healthy gastric tissue), and data-processing pipelines. Furthermore, confounding variables such as geographic diversity, *H. pylori* status, antibiotic exposure, and dietary patterns complicate microbial profile interpretations. These inconsistencies contribute to variable outcomes and hinder cross-study comparisons.

Another critical barrier is the current reliance on observational data, which precludes definitive conclusions about correlative versus causal relationships between microbiome alterations and clinical outcomes. In order to determine the correlative versus causal nature of these microbial signatures in AGC, future studies should utilize shotgun metagenomics in longitudinal cohort studies to affirm microbial alterations, with analysis of their respective functional gene profiles that precede advanced or nonresectable staging of GC [90]. As shown in Table 2, metagenomic profiling provides a higher level of resolution of taxonomic differences and is capable of generating comprehensive functional gene profiles which may effectively differentiate GC causative vs. correlative microbial signatures. A downfall of metagenomics, however, is its increased cost compared to 16S rRNA sequencing, a cost-efficient technique more standardized in identifying biomarkers but with lower taxonomic resolution and limited in inferring biomarker function [91]. Follow-up studies may include attempts to induce pro-/anti-tumorigenic states in animal models with key microbial signatures derived from shotgun metagenomics to determine targetable pathways with therapeutic implications. Furthermore, there is a lack in distinction between altered versus discriminatory signatures in GC, as the former likely reflect significant compositional changes over time through Correa’s cascade but nonspecific to staging. Follow-up studies should apply backward stepwise algorithms or random forest models to clearly identify GC discriminatory signatures, potential novel biomarkers that may be utilized for the risk stratification and early diagnosis of GC.

Additionally, therapeutic strategies aimed at microbiome modulation with oral probiotic administration represent compelling approaches to augmenting treatment efficacy and minimizing adverse effects in AGC management.

## 6. Conclusions

Key biomarkers within the gastrointestinal microbiome hold significant promise for improving the early diagnosis, risk stratification, and personalized treatment of GC. Given the typically late-stage diagnosis of GC and its associated poor prognosis, identifying targetable and diagnostic microbiome-derived indicators is critical. Although *H. pylori* infection confers the highest risk of GC, *F. nucleatum* is also observed to be consistently enriched in GC states across multiple studies. Furthermore, its activation of the pro-inflammatory IL-17/NF-κB pathway presents a targetable pathway in the treatment of GC. Contrarily, recent studies elucidate the robust anti-tumor properties of *Bifidobacterium* sp. and *L. rhamnosus GG* through modulation of the cGAS/STING signaling pathway, directly activating DCs and CTL activity. Diagnostic markers for GC include enriched *Streptococcus* and *Lactobacillus* in gastric/stool samples, and depleted *Helicobacter* in stool samples; however, these identifiers remain inconsistent between studies with multiple confounding variables, including geographic differences, sample type, and statistical analysis.

With current evidence supporting the microbiome’s role in GC pathogenesis and modulating host immunity, microbial biomarkers should be integrated into comprehensive diagnostic and treatment frameworks to identify at-risk individuals and optimize treatment responses. Future research should prioritize representative large-scale longitudinal cohort studies with standardized sequencing techniques to identify significantly altered genera in GC with consideration of shotgun genomics to determine species/strain-specific taxa and their respective functional gene profiles. Subgroup analysis may identify demographic-specific variations in microbial signatures, critical to personalized risk stratification and treatment of GC when considering probiotic administration. Additionally, prospective human-based trials should aim to compare patient outcomes when supplementing *Bifidobacterium* and *L. rhamnosus GG* in patients initiating treatment with ICIs plus CT compared to CT alone.

## Figures and Tables

**Table 1:** Studies identifying significantly altered/diagnostic microbial signatures in GC.

Author, Year	Study Type	Sequencing Method/Sample Type	Ethnic/Racial Group(s)	Altered Markers in GC	GC Discriminatory Markers
Wang et al., 2024 [9]	Meta-analysis	16S rRNA/gastric tissue (*n* = 1642) Fecal sample (*n* = 394)	China, South Korea, Colombia	Enriched: *Streptococcus, Lactobacillus* Depleted: *Roseburia, Faecalibacterium, Phascolarctobacterium*	Enriched: *Streptococcus, Lactobacillus*
Abate et al., 2022 [10]	Two-cohort retrospective analysis	IMPACT assay (hybrid capture-based NGS platform)/gastric tissue (*n* = 520)	White, Asian, Black	Enriched: *Streptococcus, Prevotella, Lactobacillus, Helicobacter*	Not reported
Liu et al., 2022 [5]	Meta-analysis	16S rRNA/gastric tissue (*n* = 825)	China, South Korea, Malaysia, Portugal, Mexico	Enriched: *Fusobacterium, Prevotella, Streptococcus, Stenotrophomonas*, *Lactobacillus* Depleted*: Bifidobacterium, Halomonas, Shewanella, Helicobacter, Bacillus,* *Blautia*	Enriched: *Dialister, Granulicatella*, *Comamonas, Chryseobacterium* Depleted: *Helicobacter*
Zhou et al., 2022 [8]	Multi-center observational study	16S rRNA/paired gastric tissue and fecal sample (*n* = 1043)	China	Enriched: *Streptococcus anginosus, Streptococcus constellatus*	Not reported
Coker et al., 2018 [6]	Observational cohort	16S rRNA/gastric tissue (*n* = 81)	China	Enriched: *Fusobacterium nucleatum, Prevotella intermedia*, *Prevotella oris, Peptostreptococcus stomatis, Dialister pneumosintes,* *Slackia exigua, Catonella morbi*	Not reported
Yang et al., 2016 [11]	Cross-sectional observational	16S rDNA/gastric tissue (*n* = 40)	Colombia	Enriched: *Leptotrichia wadei* and *Veillonella* sp.	Not reported

**Table 2:** Comparison of 16S rRNA sequencing and shotgun metagenomic sequencing in GI microbiome characterization.

Characteristics	16S rRNA Sequencing	Shotgun Metagenomic Sequencing
Target	Bacterial 16S rRNA gene	Whole genome (DNA)
Resolution	Genus	Species/strain
Sample type	Gastric tissue biopsy (low biomass)	Fecal sample (high biomass)
Costs	USD ~50–100	USD ~100–1000
Advantages	Effective for large-scale targeted studies, cost-efficient, standardized pipelines, biomarker discovery	Comprehensive genome characterization, gene function profiling, increased taxonomic resolution, broad microbe detection
Disadvantages	Primer bias, poor functional insight, limited taxonomic resolution, restricted to detection of bacterial microbes	Expensive, host DNA contamination, less established pipelines

## Data Availability

No new data were created or analyzed in this study. Data sharing is not applicable to this article.
